# Supplementary material for: Content-rich biological network constructed by mining PubMed abstracts
Source: BMC Bioinformatics. 2004 Oct 8;5:147. doi: 10.1186/1471-2105-5-147 (PMC528731; doi:10.1186/1471-2105-5-147)
Supplement: Additional File 2 — The original results of the above study (non-essential files are deleted to keep the file size under the limit set by BMC bioinformatics). [file 1471-2105-5-147-S2.bz2 › chilibotAdditionalFile2/dip30/663ID9450761E11056/html/NFKB1_RELA.html]

 


 **NFKB1** and **RELA** 
  
Found 53 abstracts in PubMed, retrieved 30.  
 

 What does Google say? 
 PDF only 
| .edu only 

---

**Interactive relationship** (e.g. stimulation, inhibition, etc)

**Neutral relationship**- In addition, NF kappa B p65  [ **RELA** ]   **RelA** , but not NF kappa B p50  **NFKB1** , binds specifically to the NF kappa B site.  Ref: 8207232 J Immunol, 1994
- Immunosupershift and UV cross linking established that the keratinocyte nuclear binding protein of site B 5 GGGGCTTTCC 62 to 253 bp was NFkB  [ **NFKB1** ]  consisting of the p65  [ **RELA** ]  and p50 subunits.  Ref: 7525574 J Biol Chem, 1994
- In comparison to the conventional GC box containing Sp1 motif, the alpha V kappa B motif also binds in vitro to c Rel and  **RelA**  but not to  **NFKB1** .  Ref: 8572570 Anticancer Res< MedlineTA>Anticancer Res, 1994
- In extracts from activated murine macrophages and macrophage cell lines this element binds several non canonical NF kappa B Rel complexes, in addition to p50  **NFKB1**  homodimer and p50 p65  [ **RELA** ]  NKFB1  **RelA**  heterodimer.  Ref: 7624137 Oncogene, 1995
- Our previous work demonstrates that the Rel NF kappa B family proteins c Rel,  **RelA**  p65  [ **RELA** ] , and  **NFKB1**  p50 are involved in the complex that binds to the CD28RE.  Ref: 7623820 Mol Cell Biol, 1995
- Further characterization has revealed the activation and interaction of C EBP alpha, C EBP beta NF IL6,  **NFKB1**  p50, and  **RelA**  p65  [ **RELA** ]  to their specific binding elements present in the bovine IL 6 gene.  Ref: 7669256 DNA Cell Biol, 1995
- Nuclear protein binding to the NFkB  [ **NFKB1** ]  binding sequence was altered by antibodies to the p50 and p65  [ **RELA** ]  components of NFkB  [ **NFKB1** ] .  Ref: 8554599 Biochem Biophys Res Commun, 1995
- One nucleoprotein complex contained prototypical NFKB  [ **NFKB1** ]  comprising of p50 and p65  [ **RELA** ]  subunits and a second contained the p65  [ **RELA** ]  subunit and an 80 K factor, p80.  Ref: 8074713 Biochem Biophys Res Commun, 1994
- We show that selective inhibition of  **RelA**  NF kappaB decreased induction of  **NFKB1** , interleukin 2, and interleukin 2Ralpha genes but not c myc.  Ref: 9417120 J Biol Chem, 1998

**Non-interactive relationship** (e.g. studied together, co-existance, homology, etc.)

- MS association was excluded for the  **NFKB1**  and NFKB3  [ **RELA** ]  genes, which show remarkably low degrees of polymorphism.  Ref: 12058256 Genes Immun, 2002
- Antisense inhibition of the  **RelA**  subunit but not the  **NFKB1**  subunit of NK kappa B transcription factor results in a block of cellular adhesion and inhibition of tumor cell growth in vitro and in vivo.  Ref: 8572570 Anticancer Res< MedlineTA>Anticancer Res, 2002
- The active complex is composed of two subunits designated  **NFKB1**  and  **RelA**  formerly called p50 and p65  [ **RELA** ] , respectively.  Ref: 8687102 Anticancer Res< MedlineTA>Anticancer Res, 2002
- We showed that CD28 costimulation accelerated the kinetics of nuclear translocation of c Rel and its phosphorylated form, p50  **NFKB1** , and p65  [ **RELA** ]   **RelA** .  Ref: 7969133 Mol Cell Biol, 1994
- A combination of  **RelA**  p65  [ **RELA** ]  and  **NFKB1**  p50 also activated the IL 2 promoter through the CD28RE site.  Ref: 7623820 Mol Cell Biol, 1995
- Purification of this DNA binding complex demonstrated that NF kappaB is a heterodimer composed of two subunits,  **NFKB1**  and  **RelA** .  Ref: 9116500 Protein Expr Purif, 1997
- Binding sites for the NF kappa B transcription factor complex, composed of two subunits, p50  **NFKB1**  and p65  [ **RELA** ]  rel A, are present in many cell adhesion molecules, cytokines, and growth factor receptors.  Ref: 7727726 In Vivo< MedlineTA>In Vivo, 1997
- We now show that a series of topoisomerase poisons actinomycin D, camptothecin, daunomycin and etoposide also activate NF kappaB  **NFKB1**   **RelA**  dimer in ACH 2 and CEM cells.  Ref: 8932379 Nucleic Acids Res, 1996
- Over a 24 hr period of cytokine stimulation the p50 p65  [ **RELA** ]  complex of NFKB  [ **NFKB1** ]  was present in the nucleus whilst the second complex declined in abundance after two hours due to the decline of p80.  Ref: 8074713 Biochem Biophys Res Commun, 1994
- Antisense inhibition of the  **RelA**  subunit of NF kappa B transcription factor but not the  **NFKB1**  subunit causes pronounced inhibition of tumor cell growth in vitro and in vivo.  Ref: 8615671 Anticancer Res< MedlineTA>Anticancer Res, 1994
- LY294002, PI3K DN, or AKT DN inhibited NFkB  [ **NFKB1** ]  activation, p65  [ **RELA** ]  transactivation, and cyclooxygenase 2 COX 2 expression induced by lauric acid or constitutively active CA TLR4.  Ref: 12865424 J Biol ChemJ Biol Chem, 1994
- The gel supershift assay with  **Nfkb1** ,  **Rela**  and or Rel antibodies revealed that the specific molecular forms of NF kappaB activated by radiation in the spleen were  **Nfkb1**  homodimers and  **Nfkb1**   **Rela**  heterodimers.  Ref: 10360790 Radiat Res, 1999
- Using antibodies, we demonstrated that these complexes contain NFkappaB subunits  **NFkB1** , NFkB2,  **RelA**  and c Rel.  Ref: 10773315 Virus Res, 2000
- We demonstrate that  **RelA** , either as a homodimer or a heterodimer with some other members of the Rel family and not the classical NF kappa B  **RelA**   **NFKB1** , is involved in the differential growth control of tumor cells.  Ref: 8615671 Anticancer Res< MedlineTA>Anticancer Res, 2000
- Antisense phosphorothioate oligomers to  **relA**  but not  **NFKB1**  caused a rapid inhibition of cell adhesion in diverse cell types.  Ref: 8687102 Anticancer Res< MedlineTA>Anticancer Res, 2000
- To investigate whether transcription factors of the NF kappa B family could play a role in early mammalian development, we have analyzed the expression of  **nfkb1** , nfkb2, c Rel,  **RelA** , RelB, and bcl 3 from 6.5 to 10.5 day mouse embryo implantation sites.  Ref: 10402669 Dev Genet, 1999
- Although apigenin did not inhibit TNF alpha induced nuclear translocation of NF kappa B p50  **NFKB1**  p65  [ **RELA** ]   **RelA**  we found this flavonoid did inhibit TNF alpha induced beta galactosidase activity in SW480 cells stably transfected with a beta galactosidase reporter construct driven by four NF kappa B elements, suggesting an action on NF kappa B transcriptional activation.  Ref: 7543732 Am J Pathol, 1995
- In bone marrow, an NF kappaB like binding factor was induced that may be  **Nfkb1**   **Rela**  and Rel  **Rela**  like heterodimers, but it exhibited a higher mobility than  **Nfkb1**  homodimers.  Ref: 10360790 Radiat Res, 1999
